# Yi-Gang Xu: the Earth's deep interior holds the key to habitability

**DOI:** 10.1093/nsr/nwab018

**Published:** 2021-02-01

**Authors:** By Jin Liu, Ho-Kwang Mao

**Affiliations:** Center for High Pressure Science and Technology Advanced Research (HPSTAR); Professor and the Director of HPSTAR

## Abstract

The deep Earth is the engine of whole Earth systems and plays a key role in surface evolution and geological hazards. Scientists have been deciphering the internal processes that shape our habitable planet, especially since the formulation of plate tectonics theory. To date, how the deep Earth works remains mysterious. At the end of 2020, the Chinese Academy of Sciences (CAS) started to set up the Center for Excellence in Deep Earth Science, headquartered in the Guangzhou Institute of Geochemistry (GIG), with long-term support for these emerging and interdisciplinary research areas. *NSR* recently talked to Professor Yi-Gang Xu, GIG’s Director, about why the study of the Earth's interior is essential, the current progress of deep Earth science in China, and what makes our planet habitable.

## IMPORTANCE OF EARTH’S INTERNAL PROCESSES

***NSR:*** Why does the deep Earth matter?

***Xu:*** Earth is the only habitable planet in the Solar System due to two unique characteristics. First, Earth has an active interior, featuring vigorous convection of the mantle and liquid outer core. It is perhaps the core convection that generates and maintains the geomagnetic field, which is so important to the origin and evolution of life. The geomagnetic field forms an invincible barrier blocking solar winds and high-energy particle attacks. At the same time, it prevents the atmosphere from escaping. Nowadays, Earth is middle-aged in terms of its energy budget, whereas the Moon ‘died’ about 3.0 billion years ago. Mars is in its final ‘dying’ stage, and its residual energy is insufficient to maintain its full operation.

Second, although nearly all planetary bodies undergo tectonics giving birth to volcanoes, mountains, craters and faults on their surfaces, Earth appears to be the only planet with tectonic plates in the Solar System. Over the past 50 years, geoscientists have made great achievements in understanding our planet's composition, structure and dynamics. The discovery of continental drift and the consequent establishment of plate tectonics theory is definitely one of the most significant scientific breakthroughs of the 20th century. Like quantum mechanics in physics and genetic codes in biology, plate tectonics has revolutionized Earth science. In principle, plate tectonics is elegantly simple and has fundamentally transformed our concept of how the world works. It explains the movements of the Earth’s surface, typically ∼100 km thick, providing a uniform framework for decoding the processes of spreading at ocean ridges, mountain building, earthquakes, volcanoes and mineralization at convergent boundaries. It is highly relevant to human society with regard to atmospheric evolution and climate change over billions of years. More importantly, new discoveries indicate that plate tectonics has played an essential role in setting up life on Earth.

**Figure:**
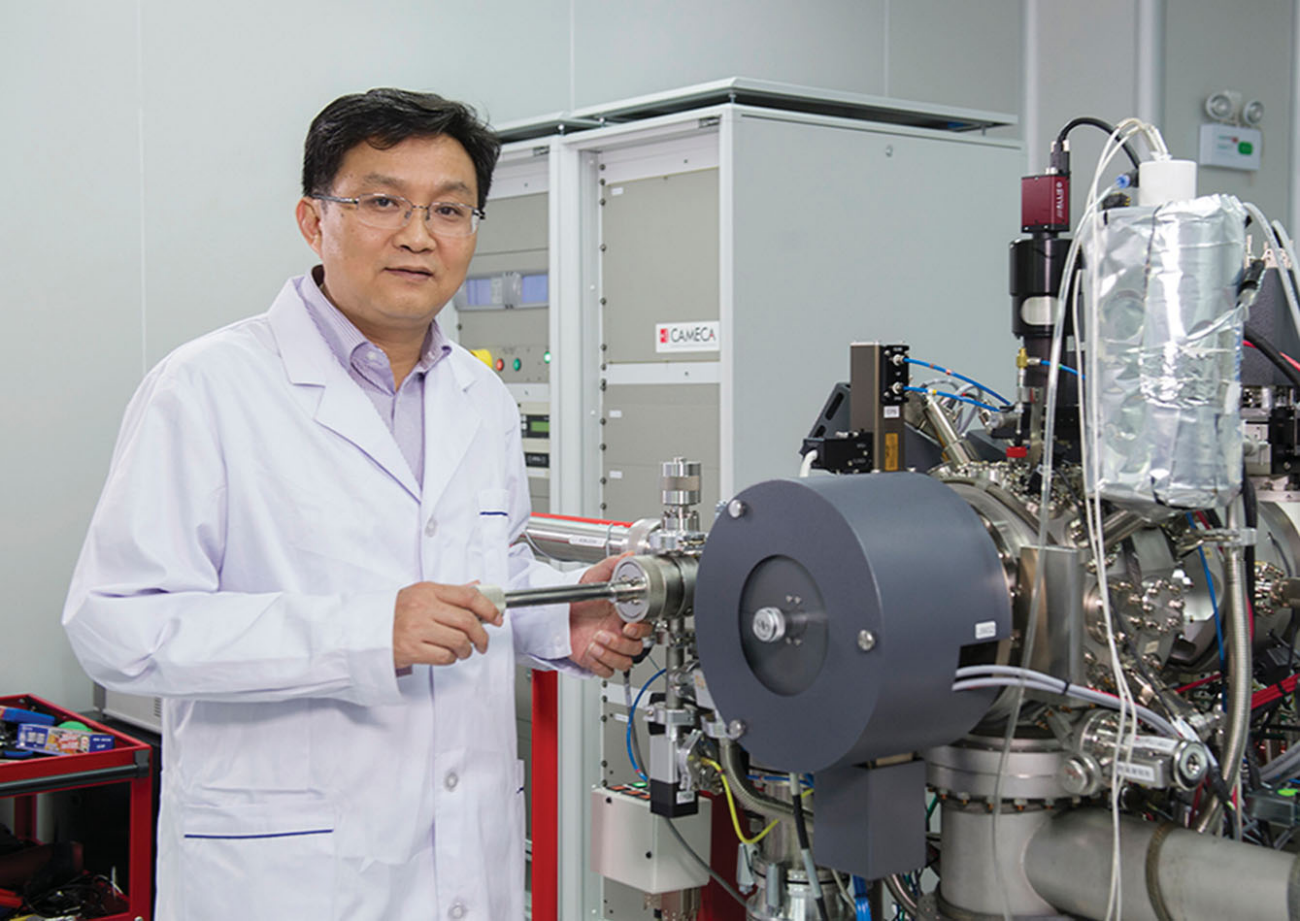
Dr Yi-Gang Xu in the lab *(Courtesy of Yi-Gang Xu)*.

Although plate tectonics is limited to our planet's uppermost layer (∼100 km, in considering a radius of 6300 km), the ultimate driving force lies in its deep interior, such as mantle convection. In this sense, understanding plate tectonics and Earth's deep interior is indispensable in understanding what makes our planet habitable.

***NSR:*** Can you provide an example of how deep processes affect Earth's habitability?

***Xu:*** I would like to take large-scale volcanism as an example because volcanoes originate from the deep Earth, and their eruptive materials such as ashes, gases and debris can penetrate diverse surface spheres such as the hydrosphere, biosphere, cryosphere and atmosphere. Consequently, volcanism can be regarded as a vital link between the geodynamic processes in Earth's deep interior and surface system evolution. There are three principal types of volcanism on Earth: mid-oceanic ridge volcanism, subduction-related arc volcanism and intraplate volcanism, in the descending order of eruption volume. The last type of volcanism is emphasized here, despite its subordinate volumes throughout Earth's entire history. If not all, many of these intraplate volcanoes are related to hot spots. They are created by high-temperature mantle plumes that exist below the tectonic plates, probably rooted in the core-mantle boundary region. The Permian Emeishan Flood Basalt in southwest China is such a typical example. It appeared ∼260 million years ago and covered an area of over half a million square kilometers. About 8 million years later, another giant continental flood basalt province, the Siberian Traps, erupted over a very short period between tens of thousands of years and hundreds of thousands. This province covered an area of >2 million square kilometers and produced 4 million cubic kilometers, corresponding to an extremely high rate of eruption. Such devastating volcanic eruptions released countless greenhouse and harmful gases into the atmosphere, significantly increasing Earth's surface temperature (∼10°C and ∼4°C by Siberian and Emeishan Traps, respectively) and affecting other parameters that are critical to habitability. A series of processes resulted in the most severe biotic crisis in the Phanerozoic. Over 90% of the oceans’ species, about 70% of vertebrate families on the land, and most land vegetation died off by the end of the Permian ∼250 million years ago.

Like quantum mechanics in physics and genetic codes in biology, plate tectonics has revolutionized Earth science.

—Yi-Gang Xu

***NSR:*** This is the largest mass extinction in Earth's history as we know it. Life almost came to an end at that time. In today's world, what is the significance of deep Earth knowledge from a social perspective?

***Xu:*** From a social perspective, the human species depends on mineral and energy resources. Industrial civilization has boomed since geoscientists have been able to discover and exploit these natural resources on a large scale. Mineral resources hatch the tools that allow society to provide manufactured goods and fuel to billions of people. The increasing population continually puts more pressure on resource supply. Understanding how the deep Earth works is critical for natural resource exploration and management. In particular, understanding fluid movement within the deep solid Earth can reveal the formation mechanisms and geological characteristics of available resources. Nowadays, geoscientists view the Earth as a whole system and integrate geology, oceanography, hydrology, atmosphere and many other disciplines into the Earth system. The deep Earth lies in our blue planet's center, and has had evolving interactions with the rest of the Earth system over the past 4.5 billion years. Earth's internal processes ultimately modulate most resources, where plate tectonics and plume tectonics provide a basis for understanding the origins and location-scale distributions of mineral and energy deposits. For example, several hundred million tons of copper have been exhumed from plate boundaries around the circum-Pacific region. The world's largest platinum group element (PGE) deposits and Ti-V deposits are respectively associated with the Bushveld Complex in South Africa and Emeishan large igneous province, both of which are mantle plume-related.

## DEEP EARTH SCIENCE IN CHINA

***NSR:*** What are the goals of deep Earth research and exploration in China?

***Xu:*** The goals of deep Earth research and exploration in China are two-fold. On the one hand, it is highly relevant to meet national demands. China's rapid economic development requires a sustainable supply of diverse resources. However, while resource exploration depths in major ore countries now frequently exceed 2000 m, most ore exploration depths in China are <500 m. Maintaining sustainable economic development in China requires the prospection of more resources from the deeper crustal level. High-resolution visualization of the deep lithospheric structure and effective prospecting technology are crucial to meet this national demand.

On the other hand, deep Earth research offers potential breakthroughs in Earth science. As I mentioned previously, while the plate tectonics theory is one of the great scientific breakthroughs of the 20th century, it nevertheless only deals with the dynamics of the superficial layer, which is <100 km thick. Although it is known that the driving force of plate tectonics is in the Earth's deep interior, how it operates remains mostly unknown. More knowledge about the structure, composition and dynamics of the deep interior will certainly help refine Earth science theory or even establish a new theory.

***NSR:*** Our understanding is only skin-deep. Steady thermal convection in the Earth's mantle cannot explain the rifting of supercontinents. We need additional mechanisms. So, what are we embracing in the post-plate tectonics era?

***Xu:*** Following demands for a new science of the Earth, Earth System Science emerged as an ambitious inter- and transdisciplinary collaborative effort in the 1980s. A major success is global change studies, involving the integration of many different disciplines of atmospheric physics and chemistry, physical oceanography, climatology and paleo-environmental reconstruction. We are looking forward to a significant understanding of the interaction between multiple spheres. Nevertheless, the deep integration of the Earth System Science framework is one of the great challenges of our time. The whole of Earth's interior must be included in the Earth system, especially when geological processes through time are taken into account. While

More knowledge about the structure, composition and dynamics of the deep interior will certainly help refine Earth science theory or even establish a new theory.

—Yi-Gang Xu

the deep Earth is presumably the engine of the Earth system as a whole, it is not yet clear to us how it works and how Earth's deep processes govern the surface system evolution. Considering the prevailing challenges in Earth System Science, we launched a multi-disciplinary strategic priority research program in 2016 with support from the CAS. Aiming to understand how Earth's surface systems interact with its interior processes, the program focuses on the three major aspects of Earth's deep interior: the state of the deep Earth, its internal processes, as well as their connection with Earth's surface systems.

***NSR:*** For the past five years (2016–2020), the study of Earth's interior has been one of the Strategic Priority Research Programs (Category B) of CAS—‘linking the Earth's interior processes and surface system evolution’—and it is led by GIG. Could you tell us a bit more about this program?

***Xu:*** We are grateful to the CAS headquarters for funding this Strategic Priority Research Program. In my opinion, this represents a major step towards promoting deep Earth exploration in China. The program consists of three sub-projects. Project one aims to probe the constituents and structural heterogeneity of the Earth's deep interior through multiple approaches. These include seismology, mineral physics, computational geochemistry, isotope geochemistry, geo- and paleomagnetism, and comparative planetary science. Project two aims to better understand the interactions between Earth's interior spheres and the surface, and its recycling processes, through plate subduction and mantle plume. Special attention will be paid to the distribution and transportation of volatiles in different spheres and their influences on the physio-chemical properties of the deep mantle and deep dynamic processes. The last project is designed to assess how Earth's surface systems respond to its deep interior processes, especially in terms of the climatic and oceanic changes and their roles in the evolution of life.

This year, we are close to concluding the program and summarizing our major achievements. Firstly, our seismic imaging capability has significantly improved, and we are now quantifying the small-scale structural heterogeneity of the deep Earth. Interestingly, the roughness of the 660-km boundary is not equally distributed at the bottom of the mantle transition zone. In other words, there are massive mountains not only lying on the Earth's surface but also hidden inside the Earth. Secondly, about 200 meteorite impact craters have been found on Earth so far. One, the Xiuyan crater, is in the Liaodong Peninsula of China. Here, Dr Ming Chen at GIG discovered natural diamond formation from reactions between CO_2_ and the dominant mantle oxide (Fe, Mg)O. Last but not least, multiple efforts have

Although it is known that the driving force of plate tectonics is in the Earth's deep interior, how it operates remains mostly unknown.

—Yi-Gang Xu

**Figure:**
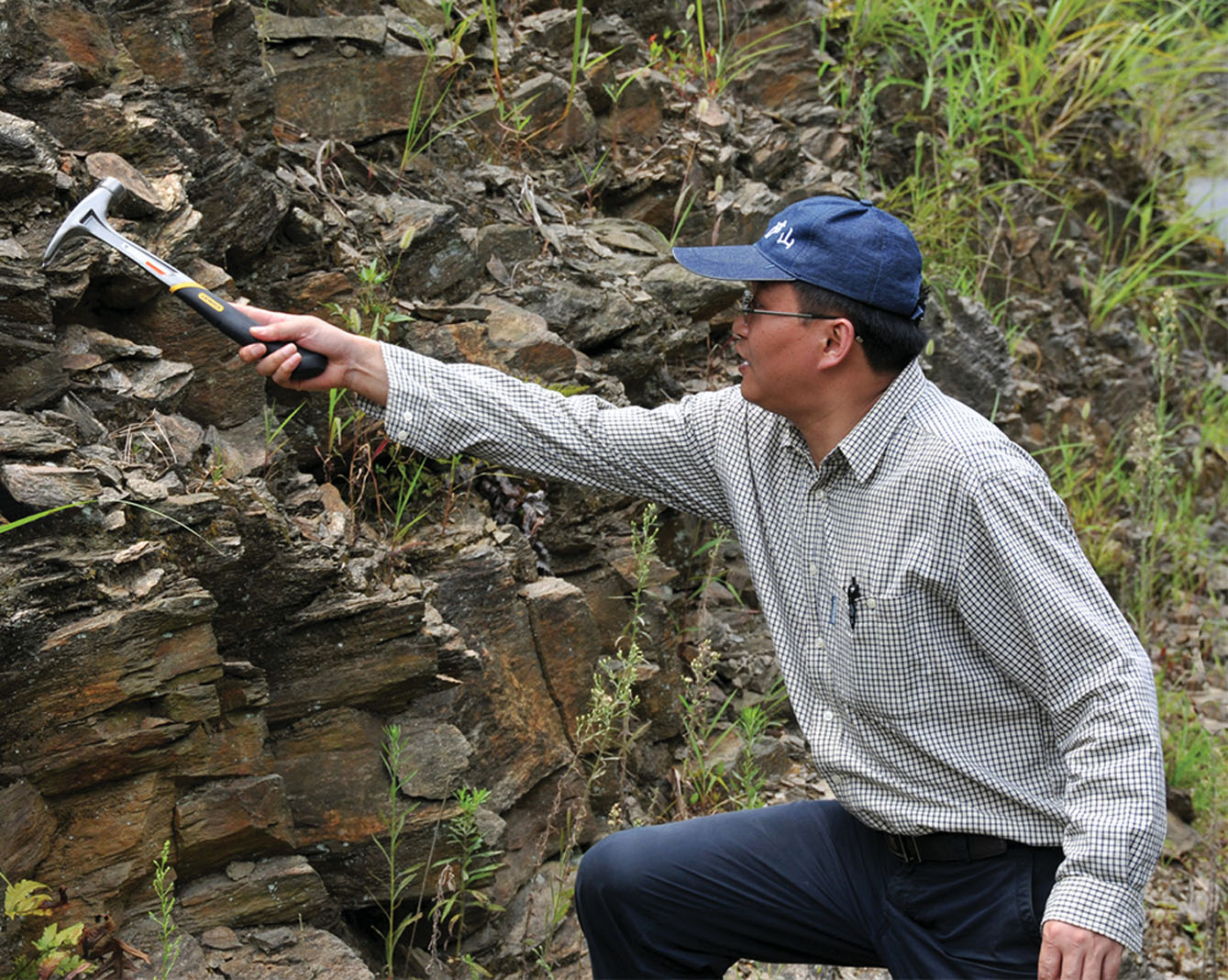
Dr Yi-Gang Xu in the field, South China *(Courtesy of Yi-Gang Xu)*.

focused on the causes of the Permian-Triassic boundary (PTB) extinction when nearly 90% of the planet's species were killed. We found that large-scale volcanism and rapidly enhanced continental weathering might have delivered excessive nutrients to oceans, causing marine eutrophication, anoxia, acidification and ecological perturbation, and thus, might ultimately have led to the end-Permian mass extinction.

***NSR:*** In addition to scientific progress, what are the main benefits of the operation of this program?

***Xu:*** From a managerial point of view, two points are worth mentioning. The first concerns cross-disciplinary interaction: the CAS strategic priority program brings together two different teams that had rarely worked together before, one focusing on the deep interior and the other focusing on the surface system. The collaboration and interaction between these two groups yield some very exciting results and provide new perspectives on Earth system science theory development. The second is that such a program's operation considerably enhances our ability in geophysical exploration, geochemical tracing, geochronological dating and experimental and computational modeling. Both the new philosophy of research and the instrumentation of relevant analytical platforms and simulation devices have considerably promoted deep Earth research in China and have significantly advanced our understanding of the physics, chemistry and dynamics of Earth's interior.

I am delighted that this progress has been recognized by the CAS and the National Natural Science Foundation of China (NSFC). In particular, we are setting up the CAS Center for Excellence in Deep Earth Science in Guangzhou. Meanwhile, CAS and NSFC have co-funded strategic research from 2021 to 2035 on Earth's internal processes and habitability. With the help of these new programs, we aim to explore the history of Earth's deep interior in detail as an example of a habitable planet, and from that history, try to deduce the likelihood of similar histories occurring elsewhere in the Universe.

## BLUE EARTH AND PLANETARY HABITABILITY

***NSR:*** So what makes our planet habitable?

***Xu:*** Among all the factors that make a planet habitable, two common substances, carbon dioxide and water, set the tone and play a central role. Our blue planet has evolved not only with the atmosphere, hydrosphere and biosphere but also with the Earth's interior. The origin and evolution of life could not exist on Earth if there were no stable atmosphere and hydrosphere. In the Solar System, the atmosphere of Venus is made up almost entirely of carbon dioxide and is 100 times thicker than that of Earth. Carbon dioxide is second only to water in its greenhouse capacity, making the Venusian surface too hot for life, reaching as high as 470^o^C. Life seems limited to a temperature range of −15 to about 200^o^C. Low temperatures cause chemicals to react slowly, interfering with the reactions necessary for life; high temperatures can break apart protein and genetic materials.

Carbon dioxide plays an essential role in establishing the surface temperature of Venus. Fortunately, there is very little carbon dioxide currently in the Earth's atmosphere, while the vast majority of carbon has been locked up in the solid Earth. If all Earth's carbon were in the form of gaseous carbon dioxide, Earth's atmospheric pressure could increase by a factor of ∼100. Interestingly, this is comparable with that of Venus, implying that Earth and Venus might have started with almost the same volatile ingredients. Thus far, calcium carbonate is the largest reservoir of carbon dioxide in the Earth's crust. In general, carbonate minerals are more stable than hydrous minerals in subducting plates, and a large amount of carbon could have descended into the deep Earth. To date, the overall carbon budget related to outgassing and subduction through the interaction with Earth's interior remains to be understood.

***NSR:*** Calcium carbonate is the main component of seashells on the beach. It is interesting to know that small seashells can lock up a huge amount of carbon dioxide, preventing Earth's surface from overheating. In addition to carbon dioxide, what does water contribute to the habitability of our blue planet?

***Xu:*** Water is not as volatile as carbon. Earth-like life needs liquid water as we know it. Much organic matter contains water and undergoes transformations associated with hydration and dehydration. Remember that the human body consists of 60% water, and 71% of our planet's surface is covered by water. As a matter of fact, water might have been present prior to life on Earth and life most likely adapted to involve the abundance of water. The oldest sediments found in Greenland show that water was present as early as 3.8 billion years ago at least. Ancient zircons hold essential clues to the early presence of liquid water as far back as 4.4 billion years. Notably, changes in the sea level can be employed to delimit the surface water budget. The amount of water on the Earth's surface could have remained within a tight range over billions of years, meaning that ocean water on Earth is a balance between outgassing and ingassing water. Water–rock interactions can cause an alteration of the crust to produce water-bearing or CO_2_-bearing minerals. Through these volatile-rich materials’ subduction, volatiles are returned into Earth's deep interior, which balances the volcanic flux to the atmosphere.

It appears that a stable climate that keeps liquid water on the surface is most important for life. It is in part determined by the physics, chemistry and dynamics of the Earth's deep interior. Ultimately, we only know a fraction of what makes our blue planet habitable; lots of mysteries are waiting to be explored and solved.

Ultimately, we only know a fraction of what makes our blue planet habitable; lots of mysteries are waiting to be explored and solved.

—Yi-Gang Xu

***NSR:*** Answers to some of these mysteries are hidden in the depths of the Earth. Linking the Earth's internal processes and habitable environments is crucial. What key research directions do you think we should follow in the future?

***Xu:*** In order to gain a better understanding of the link between the Earth's interior processes and habitable environments, the core questions we must ask include: what is the role of early Earth processes in shaping our planet? What triggered the major geological events through Earth's history and how did these events shape our planet's evolution? How did the volatiles cycle between the Earth's interior and surface system, and what were their roles in the evolution of life? Are novel chemical reactions at depth a new sort of Earth engine? What are Earth's internal processes, and how do they govern the formation of ore deposits, volcanic eruptions and earthquakes?

Details of these exciting unsolved problems will be summarized in a new book on the scientific strategy for the deep Earth sciences, which is due to be published in 2021.

## Notes

Jin Liu is a Professor at the Center for High Pressure Science and Technology Advanced Research (HPSTAR) and Ho-Kwang Mao is a Professor and the Director of HPSTAR.

